# The first complete plastome sequence from family Flagellariaceae (*Flagellaria indica* L., Poales)

**DOI:** 10.1080/23802359.2021.1987176

**Published:** 2021-10-07

**Authors:** Sangjin Jo, Seonghyun Cho, Wonhee Kim, Ki-Joong Kim

**Affiliations:** a Division of Life Sciences, Korea University, Seoul, South Korea; b National Institute of Biological Resources, Incheon, South Korea

**Keywords:** Plastome, Flagellariaceae, *Flagellaria indica*, Graminid clade, small inversion

## Abstract

As a part of phylogenomic study of graminids, we report the complete plastome sequence of *Flagellaria indica* L. (Flagellariaceae) (NCBI No. MZ504969). This is the first reported complete plastome sequence from the Flagellariaceae. This plastome shows typical quadripartite structure. The plastome size is 161,643 bp, which consists of 88,714 bp large single copy (LSC), 19,065 bp small single-copy (SSC), and 26,932 bp inverted repeat (IR) regions. However, we detected *F. indica* plastome has a 288 bp small inversion between *ycf*3 and *trn*S-GGA. The palindromic repeats of 10 bp (TTCCAATTTC/GAAATTGGAA) were located on the two break points of inversion. *F. indica* plastome contains 113 genes, including 79 protein-coding, 30 tRNA, and four rRNA genes. Unlike other families of graminids, the functional *ycf*1 and *ycf*2 genes exist. Sixteen genes contain one intron and two genes (*clp*P and *ycf*3) have two introns. Sixty-two simple sequence repeat (SSR) loci are scattered in the plastome, respectively. The phylogenetic tree shows that Flagellariaceae are the basal sister lineage of other graminid families.

The graminid clade of Poales consist of four families; Flagellariaceae, Ecdeiocoleaceae, Joinvilleaceae, and Poaceae (APG IV 2016). As a part of phylogenomic study of graminids, we generated a complete plastome sequence of *Flagellaria indica* Linnaeus 1753 (Flagellariaceae) (NCBI No. MZ504969). This is the first reported complete plastome sequence from the family Flagellariaceae. The family consists of a single genus and five species (Christenhusz et al. 2018). *Flagellaria indica* is distributed in tropical and subtropical regions of Asia, East Africa, Australia, and Pacific islands. Other species mainly distribute in Pacific islands (Wepfer and Linder 2014).

The dried leaves of *F. inidica* were sampled from Koh Kong city, Koh Kong Province, Cambodia (N11°36′08.01″, E103°00′36.61″). They were ground into powder in liquid nitrogen and total DNAs were extracted using the G-spin^TM^ IIp for Plant Genomic DNA Extraction Kit (iNtRON Biotechnology). The voucher specimen was deposited in the Korea University Herbarium (KUS acc. no. TCA2009-1388) and genomic DNA is deposited in the Plant DNA Bank in Korea (PDBK acc. no. 2009-1388). The complete plastome sequence was generated using an Illumina NovaSeq platform (Illumina Inc., San Diego, CA). De novo assembly was performed using the NOVOPlasty 4.3.1 (Dierckxsens et al. 2017). Annotation was performed using the Geneious 11.1.5 (Biomatters Ltd.; Kearse et al. 2012), National Center for Biotechnology Information (NCBI) BLAST, and tRNAscan-SE programs (Lowe and Eddy 1997). The average coverage of *F. indica* plastome was 464.9x. The simple sequence repeats (SSRs) were detected with the Phobos v. 3.3.12 program (Leese et al. 2008) in the Geneious 11.1.5. For the phylogenetic analysis, we selected and downloaded 11 related complete plastome sequences based on the APG IV system (APG IV 2016) from the NCBI database.

The plastome size of *F. indica* is 161,643 bp, which consists of 88,714 bp large single copy (LSC) region, 19,065 bp small single-copy region (SSC), and 26,932 bp inverted repeat (IR) region. This shows a typical quadripartite structure. However, we detected *F. indica* plastome has a 288 bp small inversion between *ycf*3 and *trn*S-GGA. The palindromic repeats of 10 bp (TTCCAATTTC/GAAATTGGAA) were located on the two break points of inversion. This inversion is shared by the graminids group in Poales (Hiratsuka et al. 1989; Ogihara et al. 2002; Wysocki et al. 2016). The plastome holds 113 unique genes, including 79 protein-coding genes, 30 tRNA genes, and 4 rRNA genes. Six protein-coding, eight tRNA, and four rRNA genes are duplicated in the IR regions. Unlike other families of graminids, the functional *ycf*1 and *ycf*2 genes exist (Darshetkar et al. 2019). The average A-T content of the plastome is 62.8%. Sixteen genes contain one intron and two genes, *ycf*3 and *clp*P, have two introns. A total of 62 simple sequence repeat (SSR) loci are distributed throughout the *F. indica* plastome. Among these, 40, 15, and 7 are mono-SSR, di-SSR, and tri-SSR loci, respectively.

To establish the phylogenetic relationships of Flagellariaceae, we constructed a maximum likelihood tree using 12 Poales taxa. Phylogenetic analysis was performed on a data set that included 71 protein-coding genes and four rRNA genes from the 12 selected taxa using RAxML v.8.2.12 in CIPRES webserver (Stamatakis 2014) with a GTR + G + I model and 100 bootstrap replicates. The 75 gene sequences (59,754 bp in length) were aligned with the MUSCLE program using Geneious v. 11.1.5 (Biomatters Ltd.; Kearse 2012). The resulting tree supports the monophyly of graminids by 100% bootstrap value. Within the graminid clade, Flagellariaceae (*F. indica*) was a basal independent lineage and Joinvilleaceae and Poaceae from a subclade. The graminids was the sister group of xyrids (Figure 1). Restiids was suggested as a sister group to the graminids by previous researchers (Bouchenak-Khelladi et al. 2014; Linder and Rudall 2005). However, none of complete plastome sequence of restiids are available at this moment. In contrast, abundant plastome sequences are available from Poaceae. In order to establishes the comprehensive phylogenetic relationships of Poales at family level, the plastome sequences from various unexploited small families are required. Therefore, the complete plastome sequence of *F. indica* (Flagellariaceae) will provide a useful resource for the evolutionary and phylogenomic studies of Poales.

**Figure 1.**
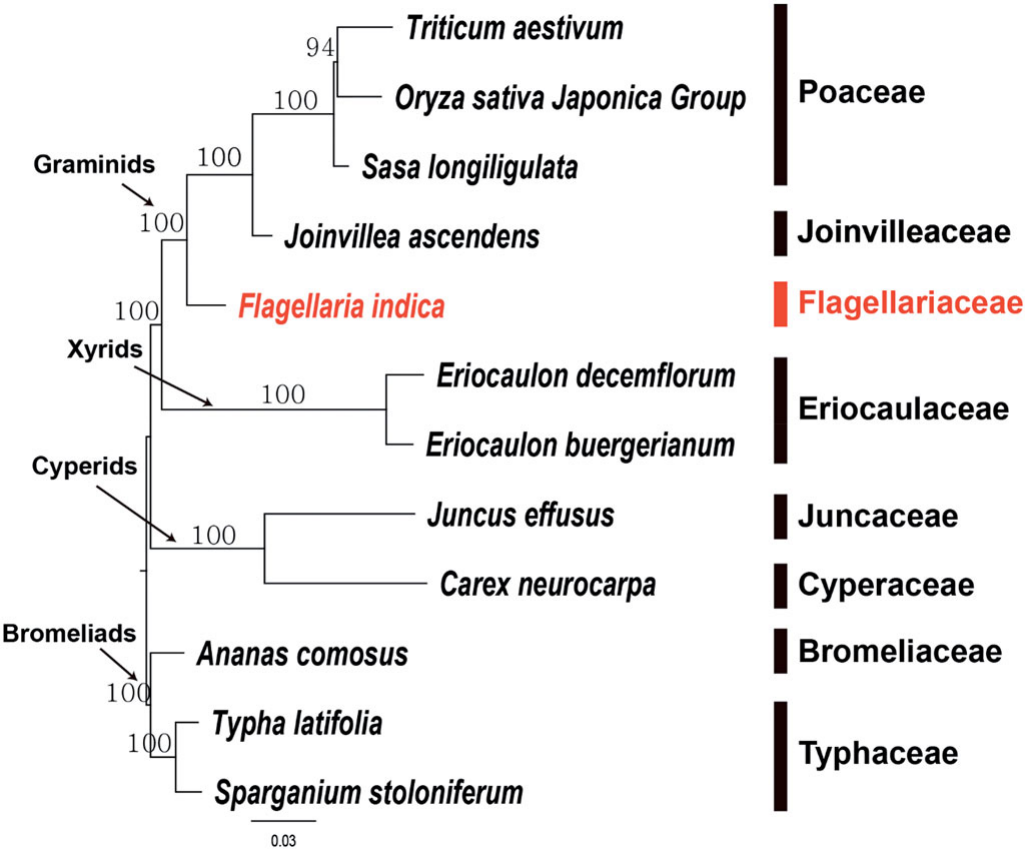
Maximum-likelihood (ML) tree based on 71 protein-coding and four rRNA gene sequences from 12 plastomes of basal Poales as determined by RAxML(-ln *L =* 212125.401397). The numbers at each node indicate the ML bootstrap values.

## Data Availability

A specimen was deposited at the Korea University Herbarium (KUS, http://mpl.korea.ac.kr/herbarium.asp, Ki-Joong Kim, kimkj@korea.ac.kr) under the voucher number TCA2009-1388 and genomic DNA is deposited in the Plant DNA Bank in Korea (PDBK, http://pdbk.korea.ac.kr/index.asp, Ki-Joong Kim, kimkj@korea.ac.kr under the DNA no. PDBK 2009-1388. The genome sequence data that support the findings of this study are openly available in GenBank of NCBI at https://www.ncbi.nlm.nih.gov under the accession no. MZ504969 for *Flagellaria indica*. The associated BioProject, BioSample, and SRA numbers are PRJNA749810, SAMN20420846, and SRR15253612, respectively.
